# Effects of exercise type and total physical activity on high-altitude acclimatization among college students

**DOI:** 10.3389/fpsyg.2026.1848841

**Published:** 2026-07-31

**Authors:** Mingli Xi, Changhao Tian, Yonghao Ou, Runqing Lu, Chen Wang, Hao Li

**Affiliations:** College of Education, Xizang University, Xizang Autonomous Region, Lhasa, China

**Keywords:** exercise therapy, high altitude acclimatization, high altitude adaptation, moderating effect, physical activity, sleep quality

## Abstract

**Background:**

Malacclimatization to the hypobaric hypoxic environment at high altitude represents a core health concern among college students residing on the plateau. Exercise serves as an important non-pharmacological strategy to improve high-altitude acclimatization; however, its underlying pathways and boundary conditions remain unclear.

**Objective:**

This study aimed to examine the effects of basketball and badminton on high-altitude acclimatization score among college students at high altitude, and to test the mediating role of sleep quality and the moderating effect of daily total physical activity, thereby providing empirical evidence for personalized exercise interventions for plateau populations.

**Methods:**

A post-test only cluster-randomized controlled design was adopted. A total of 295 sophomores living at high altitude were enrolled (146 in the basketball group and 149 in the badminton group), who received a standardized 18-week exercise intervention. Data were collected using the High-Altitude Adaptation Scale, the Pittsburgh Sleep Quality Index (PSQI), and the International Physical Activity Questionnaire (IPAQ), along with physiological measurements. After controlling for demographic and physiological covariates, statistical analyses were performed using the Mann–Whitney U test and mediation–moderation models based on Hayes’ PROCESS macro. HC3 heteroscedasticity-robust standard errors and 5,000 bootstrap samples were applied to ensure robustness of the results.

**Results:**

(1) Total physical activity significantly moderated the relationship between exercise type and high-altitude acclimatization (interaction B = −2.9965, *p* = 0.0056). The Johnson–Neyman interval test revealed that the badminton group showed significantly lower high-altitude acclimatization scores, indicating better adaptation than the basketball group only at a high activity level. (2) No significant mediating effect of sleep quality was found in the above relationship, and the prerequisite condition for mediation testing was not met.

**Conclusion:**

The effect of exercise type on high-altitude acclimatization score is moderated by total physical activity. Differences between basketball and badminton only emerge within a specific range of activity volume. Sleep quality did not mediate this pathway under the current intervention conditions. These findings reveal boundary conditions of exercise intervention effects and provide a scientific reference for college students at high altitude to select appropriate exercise modes.

## 1 Introduction

With socioeconomic development in high-altitude regions, the number of migrants to the plateau has steadily increased, and health issues related to high-altitude acclimatization have become increasingly prominent (Burtscher et al., 2025; Gatterer et al., 2024; Jia et al., 2025). Hypoxia represents the core environmental factor affecting physiological function among plateau migrants (Burtscher et al., 2025; Grover et al., 1986). Prolonged hypoxic exposure may lead to malacclimatization, manifested as a series of physiological and psychological symptoms including disrupted sleep architecture, decreased cognitive function, and reduced exercise tolerance (Madinabeitia-Cabrera et al., 2023; Hrozanova et al., 2021; Qiu et al., 2024; Shi et al., 2025). Among various intervention strategies, exercise—an effective and safe non-pharmacological approach—has been shown to promote high-altitude acclimatization by improving cardiopulmonary function and optimizing tissue oxygen utilization efficiency (Liu et al., 2024; Li et al., 2024).

However, a critical gap exists in the current literature. Most existing high-altitude exercise research has focused exclusively on acute acclimatization in newly arrived individuals, typically within days or weeks of exposure. This focus overlooks a large and growing population: migrants who have resided at high altitude for over one year. Importantly, high-altitude acclimatization is a continuous, gradual process rather than a binary endpoint. While one year of residence at 3650 m marks the completion of basic physiological acclimatization—effectively eliminating the risk of life-threatening acute mountain sickness—subclinical functional deficits (e.g., persistent sleep disturbance, reduced exercise tolerance, and chronic fatigue) often persist and can impair daily functioning for years (Liu et al., 2014; Naeije, 2010; Qi et al., 2015). These subclinical functional deficits not only significantly impair their academic performance and quality of life, but also increase the long-term risk of chronic mountain sickness. Migrant college students relocating from lower altitudes for education face significant challenges in achieving optimal acclimatization during these initial years of residence. These young migrants represent a particularly vulnerable group, as they must balance academic demands with the physiological stress of hypoxic exposure (Chen et al., 2025; Wang et al., 2024; Yang et al., 2024; Zhang D. Y. et al., 2025). Understanding how modifiable lifestyle factors—particularly exercise habits—influence their progressive functional acclimatization process is therefore of substantial practical importance, yet remains largely unaddressed (Song et al., 2024; Shi et al., 2025).

To date, high-altitude exercise research has predominantly focused on single exercise modalities, such as trekking, running, or cycling, with limited attention to comparing the differential effects of popular team sports (Li et al., 2024). Basketball and badminton are two widely practiced sports among Chinese college students, yet they differ fundamentally in their physiological loading characteristics. Basketball is characterized by sustained intermittent high-intensity activity, with frequent sprints, jumps, and physical contact, resulting in sustained cardiopulmonary demand (Albishi, 2024; Lukonaitiene et al., 2020; Deka et al., 2017). Badminton, while also intermittent, involves shorter bursts of activity with longer recovery periods and places greater emphasis on skill execution and neuromuscular coordination (de Franca Bahia Loureiro et al., 2017). Notably, these physiological load differences are amplified under high-altitude hypoxic conditions: the continuous high cardiopulmonary demand of basketball is more likely to cause oxygen supply–demand imbalance and cumulative oxidative stress, while the intermittent pattern of badminton allows for sufficient oxygen replenishment during recovery periods. These differences may translate into divergent effects on physiological acclimatization and, crucially, on sleep regulation under hypoxic conditions. This environmental amplification effect may lead to fundamentally divergent impacts of the two sports on high-altitude acclimatization.

To our knowledge, no study has directly compared intermittent high-intensity team sports with skill-based racket sports in the context of high-altitude acclimatization (Li et al., 2024). A recent review of athletic guidelines for high-altitude training concluded that existing recommendations are highly variable and lack substantial evidence to support specific sport-modality comparisons, underscoring the urgent need for head-to-head trials of this nature (Li et al., 2024).

Sleep plays a critical regulatory role in this process (Ciorciari et al., 2024; Wittmann et al., 2006). Polysomnographic studies have consistently shown that exposure to altitudes above 2,500 m leads to reduced slow-wave sleep, increased nocturnal awakenings, and disrupted sleep continuity (Hrozanova et al., 2021; Shi et al., 2025). These changes are primarily driven by hypoxia-induced alterations in brainstem neurotransmitter systems. Importantly, sleep quality is closely associated with acclimatization capacity: individuals with better sleep typically exhibit higher levels of high-altitude acclimatization (Ciorciari et al., 2023; Li et al., 2024; Liu et al., 2014). This raises the possibility that exercise may promote high-altitude acclimatization not only through direct physiological improvements (e.g., cardiopulmonary function, oxygen utilization) but also indirectly through improved sleep quality. If this cascade is validated under hypobaric hypoxia, it will provide a novel theoretical framework for combined exercise and sleep interventions to enhance plateau acclimatization.

Exercise is widely recognized as an effective non-pharmacological intervention for improving sleep quality in normoxic populations (Kelley and Kelley, 2017). The theoretical framework follows the exercise–sleep–acclimatization cascade proposed by previous normoxic studies (Kelley and Kelley, 2017), which remains untested under hypobaric hypoxia. Meta-analytic evidence indicates that regular moderate-intensity exercise significantly improves subjective sleep quality, reduces sleep onset latency, and increases slow-wave sleep duration in normoxic adults (Kelley and Kelley, 2017). However, whether these effects translate to high-altitude settings remains uncertain, as hypoxic conditions may alter the thermoregulatory and autonomic responses to exercise, potentially modifying the sleep-promoting benefits (Hrozanova et al., 2021).

Building on the above literature, three core research gaps remain unaddressed in the field of high-altitude exercise intervention. First, the mediating role of sleep quality in the exercise–acclimatization pathway has not been systematically tested under high-altitude conditions. Sleep is a strong moderator of the altitude training response, and acclimatization variability may be partly attributable to individual differences in sleep quality and recovery (Hrozanova et al., 2021). While this mediating pathway has been proposed theoretically, it has not been empirically verified under hypobaric hypoxic conditions (Li et al., 2024; Wu, 2025; Dai et al., 2026; Xu et al., 2025). Second, individual differences in habitual physical activity levels — a key factor that may influence responsiveness to structured exercise interventions — have not been adequately considered as moderating variables in existing studies. Third, head-to-head comparisons of different exercise types (e.g., team sports such as basketball vs. racket sports such as badminton) remain scarce; existing research has almost exclusively focused on single exercise modalities, which limits the evidence base for personalized exercise recommendations for high-altitude migrants who have passed the acute phase but still have subclinical functional deficits. By addressing these gaps, the present study seeks to move beyond the one-size-fits-all approach to exercise prescription for high-altitude populations.

To address these gaps, the present study implemented an 18-week cluster-randomized controlled trial comparing the effects of basketball versus badminton in college students residing at high altitude (approximately 3,650 m on the Tibetan Plateau) who had completed at least one year of residence. We aimed to test two primary hypotheses: (1) that these two exercise types have differential effects on sleep quality and high-altitude acclimatization, and (2) that individual habitual total physical activity moderates the effect of exercise type on high-altitude acclimatization. Additionally, we explored the potential mediating role of sleep quality in this relationship. By elucidating these conditional mechanisms, this study seeks to provide empirical evidence for developing personalized exercise programs tailored to high-altitude migrant populations.

## 2 Materials and methods

### 2.1 Participants

This cluster-randomized controlled study was conducted at Xizang University, Lhasa, Xizang Autonomous Region, China, located at an altitude of 3,650 m. Participants were full-time sophomores who had enrolled in either a public basketball or badminton physical education course. The selection of sophomore students was intentional, as this group had completed approximately one academic year of accumulated residence at high altitude, allowing for stabilization of basic physiological acclimatization while still being early enough in their university experience to benefit from intervention. Notably, this was the earliest scientifically valid and ethically feasible intervention window: our university strictly prohibits first-year students from participating in high-intensity competitive sports such as basketball and badminton during their entire first academic year to mitigate the risk of exacerbated acute mountain sickness and exercise-related injuries. The sample included both indigenous Tibetan students and migrant students from low-altitude regions. Ethnicity was controlled as a covariate in all analyses. To further minimize pre-intervention heterogeneity in acclimatization status, all participants had no short-term (≥24 h) exposure to altitudes above 4,500 m in the past 6 months. All participants resided continuously at 3650 m (Lhasa), and this criterion excludes only temporary travel to higher altitudes (e.g., tourism, university-assigned field missions) to prevent residual physiological perturbations from confounding the assessment of intervention effects.

Inclusion and exclusion criteria were developed based on three methodological principles: minimizing pre-intervention heterogeneity in acclimatization status, excluding confounders that affect sleep quality, exercise capacity, and psychological adaptation, and ensuring data integrity and ethical compliance. All criteria were set to align with the mediation and moderation framework of this study, as detailed below.

#### 2.1.1 Inclusion criteria

1. Full-time undergraduate sophomores aged 18–22 years with normal academic status (no leave of absence or off-campus internships); rationale: to ensure consistent exercise attendance, avoid schedule-induced measurement bias, and guarantee feasible ethical implementation.
2. Accumulated residence at the study altitude (~3,650 m) for approximately one academic year; rationale: to ensure completion of basic acclimatization and minimize pre-intervention heterogeneity in hypoxia acclimatization.
3. Regularly participated in only one target sport (basketball or badminton) for ≥1 year, ≥3 times/week, ≥60 min/session, no concurrent competitive sports; rationale: to avoid cross-contamination between sport types and isolate the effect of a single exercise modality.
4. Able to complete questionnaires and physiological measurements independently, and signed written informed consent voluntarily; rationale: to guarantee valid self-reported data and meet ethical requirements.
5. No severe organic diseases, congenital heart disease, asthma, or chronic mountain sickness; no acute infection, trauma, or surgery in the past month; rationale: to prevent pre-existing cardiopulmonary or altitude-related conditions from distorting natural acclimatization responses.

#### 2.1.2 Exclusion criteria

1. Acute respiratory infections, trauma, or postoperative recovery in the past month that may affect study outcomes; rationale: these conditions alter resting physiology and temporarily reduce exercise tolerance, confounding outcome measures.
2. Use of sedative-hypnotics, cardiovascular, anxiolytic, or anti-asthmatic medications in the past 3 months; rationale: these drugs directly interfere with sleep architecture, cardiopulmonary function, and key acclimatization indices.
3. Unhealthy lifestyle habits (heavy smoking, excessive alcohol consumption, chronic sleep deprivation); rationale: tobacco and alcohol independently impair altitude acclimatization and sleep quality, introducing additional confounders.
4. Severe missing data (>10% core items), logical contradictions, or random responses in questionnaires that could not be corrected after on-site verification; rationale: to exclude unreliable subjective data and preserve statistical validity.
5. Severe psychological symptoms requiring professional medical intervention; rationale: clinical mental disorders independently disrupt the psychological adaptation dimension of our outcome scale.
6. Course attendance rate below 85% during the 18-week intervention; rationale: insufficient training exposure cannot generate adequate exercise stimulus to reflect true intervention effects.
7. Voluntary withdrawal or incomplete data collection; rationale: missing physiological or questionnaire data preclude inclusion in mediation and moderation analyses.

A total of 303 students from eight intact classes were recruited via the university’s physical education registration system after initial eligibility screening. Cluster randomization was applied at the class level to minimize cross-group contamination and align with natural course enrollment patterns, with four classes assigned to the basketball intervention (*n* = 151) and four classes to the badminton intervention (*n* = 152). At initial data collection, participants had a mean age of 19.57 ± 1.49 years and a mean BMI of 21.84 ± 3.32 kg/m^2^. Participant screening was conducted in two standardized stages. First, on-site verification was performed immediately after data collection to identify and correct minor missing or inconsistent items with participants’ confirmation. Second, post-data-entry screening included double data entry, consistency checks, and missing value processing (samples with >10% missing core items were excluded, and a very small number of non-core missing values were appropriately imputed). During the final centralized data collection, some participants were excluded due to incomplete questionnaire responses or absence from the final physical fitness test. After data integrity screening, which excluded cases with incomplete questionnaires, missing physiological measurements, or inconsistent responses, 295 participants were included in the final valid analysis, yielding a retention rate of 97.4%.

The demographic characteristics of the basketball and badminton groups were compared to assess group comparability. Key variables included age, sex, ethnicity, height, weight, body mass index, resting heart rate, vital capacity, and blood oxygen saturation. Detailed group-specific data are presented in Table 1. As shown in Table 1, significant between-group differences were observed at data collection for sex, ethnicity, height, weight, resting heart rate, and vital capacity (all *p* < 0.05). As detailed in Section 2.4 Statistical Analysis, these variables were included as covariates in all subsequent analytical models to control for potential confounding effects.

**Table 1:** Participant characteristics (*n* = 295).

Variable	Basketball group (*n* = 146)	Badminton group (*n* = 149)	*p*-value
Demographic characteristics			
Age (years), median (IQR)	20.00 (19.00–20.00)	20.00 (19.00–20.00)	0.112
Sex, *n* (%)			<0.001
Male	94 (64.38)	58 (38.93)	
Female	52 (35.62)	91 (61.07)	
Ethnicity, *n* (%)			<0.001
Tibetan	108 (73.97)	82 (55.03)	
Non-Tibetan	38 (26.03)	67 (44.97)	
Anthropometric characteristics			
Height (cm), median (IQR)	170.00 (163.00–175.30)	165.00 (160.00–170.00)	<0.001
Weight (kg), median (IQR)	60.00 (53.00–70.00)	58.00 (50.00–67.40)	0.011
BMI (kg/m^2^), median (IQR)	21.33 (19.00–23.84)	20.82 (18.91–23.15)	0.535
Physiological characteristics			
Resting heart rate (bpm), median (IQR)	81.50 (71.00–92.00)	84.00 (73.00–94.00)	0.027
Vital capacity (ml), median (IQR)	3155.50 (2600.00–3728.00)	2772.00 (2300.00–3411.00)	0.005
Blood oxygen saturation (%), median (IQR)	90.00 (88.00–93.00)	90.00 (87.00–92.00)	0.275

IQR = interquartile range. Between-group comparisons were performed using the Mann–Whitney U test. This table presents initial participant characteristics used for covariate adjustment in all statistical models.

### 2.2 Experimental design

A post-test only cluster-randomized controlled design was employed, with exercise type as the independent variable at two levels: basketball training and badminton training. The post-test-only design was adopted because the 18-week physical education course was sufficient to produce cumulative acclimatization changes, and this design reduced participant burden and avoided measurement bias. Participants were randomly assigned to classes via the university educational administration system, with no crossover teaching interventions between groups. A single-blind design was used: the main researcher was unaware of group allocation and core study hypotheses before intervention implementation. Course instructors were not informed of group allocation or study hypotheses, and delivered only routine teaching in accordance with the university’s unified public physical education syllabus.

All procedures were conducted in the university gymnasium at an altitude of 3,650 m. The teaching period spanned September 1, 2024, to December 24, 2024, with one 90-min session per week for a total of 18 weeks. The intervention duration was selected based on previous high-altitude exercise studies that demonstrated meaningful physiological adaptations with interventions ranging from 12 to 24 weeks. The basketball and badminton groups were taught by the same instructor team, comprising three certified physical education instructors with extensive experience in both sports. This arrangement ensured that teaching qualifications and course requirements were consistent across groups, minimizing between-group confounding from non-sport-specific characteristics.

While the duration and frequency of instruction were identical, the two interventions differed fundamentally in physiological loading profiles. Basketball sessions emphasized continuous full-court running, offensive and defensive confrontation, and comprehensive fitness training, focusing on cardiopulmonary endurance and explosive power. Typical basketball sessions included 15 min of warm-up, 60 min of skill drills combined with scrimmage, and 15 min of cool-down. Heart rate monitoring in a pilot sample indicated that basketball sessions sustained heart rates in the 130–150 bpm range for approximately 70% of the active session time. Badminton sessions consisted of net-based racket swings, multi-directional footwork, and skill-based drills, with a more intermittent and technique-oriented movement pattern. Badminton sessions similarly included 15 min of warm-up, 60 min of skill drills and match play, and 15 min of cool-down, but heart rate monitoring showed a more fluctuating pattern, with peaks of 140–160 bpm during intense rallies interspersed with periods of lower intensity (100–120 bpm). To ensure consistent overall training load between groups and minimize inter-individual variation in actual exercise load beyond heart rate alone, all instructors strictly followed the unified teaching syllabus, standardizing load by fixing total session duration, training segment timing (warm-up, skill drills, match play, cool-down), exercise-to-rest ratios, and movement technical requirements. The same instructor team taught both groups to eliminate instructor-related confounding.

All data were collected in a single centralized session upon completion of the semester-long courses. Data collection occurred over a two-day period to accommodate all participants and to ensure consistency in environmental conditions. Data collection included three components: (1) standardized physiological measurements, conducted by trained research assistants using calibrated equipment; (2) standardized questionnaire assessments, including the PSQI, IPAQ-LF, and High-Altitude Adaptation Questionnaire; (3) basic demographic information. All indicators were collected on the same day in a fixed sequence: questionnaires were completed first to minimize the influence of physical exertion on subjective responses, followed by physiological measurements, with physical fitness tests conducted last. Standardized instructions were used throughout, and questionnaires were collected on-site to ensure data quality control. Accordingly, considering the observed between-group differences in the post-test only cluster design, relevant covariates were included in all analytical models to reduce potential confounding.

### 2.3 Measures

#### 2.3.1 Pittsburgh sleep quality index (PSQI)

The PSQI comprises 19 self-rated items covering seven dimensions: subjective sleep quality, sleep latency, sleep duration, habitual sleep efficiency, sleep disturbances, use of sleep medication, and daytime dysfunction (Blais et al., 1997). These dimensions are aggregated into a total score ranging from 0 to 21, with higher scores indicating poorer sleep quality. The original scale was developed by Buysse et al. (1989), with a Cronbach’s *α* of 0.83 and test–retest reliability of 0.85. In the present study, the total PSQI Cronbach’s α was 0.604, slightly below the ideal threshold of 0.70, likely reflecting the relatively homogeneous sleep patterns among healthy, acclimatized college students. The KMO value was 0.681 (*p* < 0.001), indicating that the data were suitable for factor analysis. EFA replicated the original seven-factor structure, with a cumulative variance explanation rate of 67.2%, indicating acceptable construct validity in this sample.

#### 2.3.2 International physical activity questionnaire long form (IPAQ-LF)

The IPAQ-LF was jointly developed by the World Health Organization and the International Physical Activity Association for adults aged 18–65 years (Fluetsch et al., 2019). It contains 31 items across four core dimensions: work-related physical activity, transportation physical activity, domestic and gardening physical activity, and leisure-time physical activity. Activities are converted to MET-min/week using standardized formulas (walking = 3.3 MET, moderate activity = 4.0 MET, vigorous activity = 8.0 MET), with higher total scores representing greater physical activity volume. The original scale has a Cronbach’s *α* of 0.86 and test–retest reliability of 0.82. In the present study, the IPAQ-LF total Cronbach’s α was 0.671, and the KMO value was 0.645 (*p* < 0.001). EFA replicated the four theoretical factors with 68.5% cumulative variance explained. Overall, the scale demonstrated acceptable reliability and validity for assessing total physical activity in this sample.

#### 2.3.3 High-altitude adaptation questionnaire

The High-Altitude Adaptation Questionnaire was developed by our research team with cultural and linguistic adaptation to the Xizang plateau environment (Wu, 2025; Li et al., 2024). The development process involved initial item generation based on literature review and clinical experience, followed by expert review, cognitive interviews with Tibetan college students, and pilot testing. Notably, this scale was specifically designed to assess comprehensive long-term acclimatization status, addressing the limitations of international tools such as the Lake Louise Score (LLS), which only measures acute mountain sickness (AMS) after at least 6 h of altitude exposure and lacks psychological and sociocultural dimensions (Qiu et al., 2024). The final questionnaire includes three dimensions: physiological adaptation (e.g., sleep quality at altitude, appetite changes, headache frequency), psychological adaptation (e.g., mood stability, concentration, anxiety), and social adaptation (e.g., social interaction comfort, academic engagement). Items are rated on a 5-point Likert scale ranging from “not at all” to “extremely severe,” with higher total scores indicating lower adaptation levels. The original questionnaire has a Cronbach’s *α* of 0.92 and test–retest reliability of 0.88; EFA extracts three factors with 71.3% cumulative variance explained. Its validity has been further verified using two gold-standard criteria: the Qinghai Chronic Mountain Sickness Score System (CMS) for pathological status (Wu, 2025; Li et al., 2024) and the Altitude Acclimatization Index (AAI) for physiological acclimatization in healthy populations (Wu, 2025; Li et al., 2024). Details are shown in Table 2 (adapted from Wu, 2025). In the present study, Cronbach’s α = 0.855, and Kendall’s W for construct validity was 0.420 (*p* < 0.001). To assess physiological validity, we used resting heart rate (HR) and blood oxygen saturation (SpO₂), the two most widely accepted non-invasive objective indicators of high-altitude acclimatization. Poorer acclimatization is physiologically characterized by elevated resting HR due to increased sympathetic activation and reduced SpO₂ due to impaired oxygen uptake. Using these as criteria, total scale scores were significantly positively correlated with resting heart rate (r = 0.176, *p* = 0.002), meaning participants with worse self-reported acclimatization had higher resting heart rates. The correlation between total scale scores and SpO₂ was r = 0.081, which is likely attributable to the relatively stable SpO₂ levels and low within-group variability in this sample of participants who had completed basic physiological acclimatization. This alignment between subjective questionnaire responses and objective physiological measurements supports the physiological validity of the scale. As clarified in the Introduction, the total score of the High-Altitude Adaptation Questionnaire serves as an operational measure of high-altitude acclimatization, with lower scores indicating better adjustment. The naming “High-Altitude Adaptation Questionnaire” follows the broad convention in Chinese academia, while the measured outcome strictly corresponds to reversible acclimatization as defined in international high-altitude physiology terminology.

**Table 2:** Correlations between the high-altitude adaptation questionnaire and gold-standard indicators.

Criterion (variable)	Physiological symptoms	Energy state	Emotional state	Life adaptation	Total score
Qinghai Chronic Mountain Sickness (CMS) total score	0.57^**^	0.24^**^	0.28^**^	0.04	0.37^**^
Altitude Acclimatization Index (AAI)	−0.17^*^	−0.17^*^	−0.14	−0.20^**^	−0.27^**^

Adapted from Wu (2025). *n* = 162 (independent validation sample from the Xizang Plateau). *p < 0.05, **p < 0.01. Higher CMS scores indicate more severe chronic mountain sickness; higher AAI scores indicate better physiological acclimatization.

#### 2.3.4 Physiological indicators

For physiological indicators, a Yuwell medical finger pulse oximeter (Model YX306), a Class II medical device certified by the National Medical Products Administration, was used to measure blood oxygen saturation and heart rate before physical fitness testing. Before measurement, all participants were required to rest in a quiet sitting position for 5 min. The finger clip sensor was fixed on the pulp of the index finger, and three consecutive measurements were performed with the average value recorded. The instrument was calibrated by trained research assistants before each use to ensure measurement accuracy. Vital capacity was measured using a handheld spirometer following standardized procedures: participants were instructed to inhale maximally and then exhale forcefully into the device, with the best of three trials recorded.

### 2.4 Statistical analysis

Statistical analyses were performed using SPSS 29.0 (IBM Corp., Armonk, NY, United States), with mediation and moderation analyses conducted via Hayes’ PROCESS macro (Version 5.0). A two-tailed significance level of *α* = 0.05 was adopted, and all regression models used 5,000 bias-corrected bootstrap samples to estimate 95% confidence intervals (CIs).

Shapiro–Wilk tests were first used to assess the normality of core continuous variables. Given that all core continuous variables showed significant deviations from normality (all Shapiro–Wilk *p* < 0.001, Table 3), we reported all continuous variables as median (interquartile range) and used non-parametric methods for inferential statistics: Mann–Whitney U tests for between-group comparisons and Spearman’s rank correlation for correlational analyses.

**Table 3:** Descriptive statistics and normality test results for core continuous variables (*N* = 295).

Variable	Mean ± SD	Median (IQR)	Shapiro–Wilk W	*p*-value
Total high-altitude acclimatization score	24.70 ± 7.79	23.00 (18.00–30.00)	0.927	<0.001
Total PSQI sleep score	3.96 ± 2.31	4.00 (2.00–6.00)	0.912	<0.001
Resting heart rate (bpm)	83.92 ± 13.95	84.00 (74.00–94.00)	0.965	<0.001
Blood oxygen saturation (%)	90.50 ± 4.71	92.00 (88.00–95.00)	0.931	<0.001
Vital capacity (ml)	3070.12 ± 938.61	3050.00 (2400.00–3600.00)	0.958	<0.001
Total physical activity	3444.82 ± 5256.78	2860.00 (500.00–5000.00)	0.824	<0.001
Age (years)	19.57 ± 1.49	20.00 (19.00–21.00)	0.942	<0.001
BMI (kg/m^2^)	21.84 ± 3.32	21.50 (19.50–24.00)	0.936	<0.001

IQR = interquartile range; PSQI = Pittsburgh Sleep Quality Index.

All regression models employed HC3 heteroscedasticity-robust standard errors to account for non-normality and unequal variances. To control for potential confounding, all models adjusted for eight covariates: sex, ethnicity, age, BMI, resting heart rate, height, weight, and vital capacity. These covariates were selected based on observed post-intervention group differences and established theoretical associations with high-altitude acclimatization.

Core hypothesis testing proceeded in three steps:

1. The total effect of exercise type on high-altitude acclimatization score was examined via multiple linear regression, adjusted for all covariates.
2. The moderating effect of total physical activity was tested using PROCESS Model 1, with continuous variables mean-centered. A Johnson–Neyman interval test was conducted to identify the activity range where the exercise type effect reached significance.
3. Mediating role of sleep quality (total PSQI score and seven subscales) was explored using PROCESS Model 4. This analysis was considered exploratory as the prerequisite association between exercise type and sleep quality was not statistically significant.

All models adjusted for eight covariates: sex, ethnicity, age, BMI, resting heart rate, height, weight, and vital capacity (see Table 1 for between-group differences). These variables were selected based on theoretical relevance and empirical evidence, and covered all post-intervention between-group differences to minimize confounding. Blood oxygen saturation was not adjusted for (no significant between-group difference), but was used to validate the High-Altitude Acclimatization Questionnaire. An exercise type × sex interaction term and sex-stratified subgroup analyses were conducted as sensitivity checks for sex imbalance.

Finally, univariate ANOVA comparing physiological indicators before and after covariate adjustment was performed to verify the robustness. Effect sizes are reported as unstandardized regression coefficients (B) with 95% CIs for continuous variables, χ^2^ for categorical variables, and Spearman’s rₛ for nonparametric correlations.

## 3 Results

### 3.1 Participant characteristics

A total of 295 valid participants were included in this study, comprising 146 in the basketball group (49.49%) and 149 in the badminton group (50.51%). Consistent with the initial characteristics presented in Table 1, significant between-group differences were observed for sex, ethnicity, height, weight, resting heart rate, and vital capacity (all *p* < 0.05). These imbalances are a common and inherent limitation of cluster-randomized designs, where randomization occurs at the class level rather than the individual level. These variables were adjusted for as covariates in all statistical models as described in Section 2.4.

### 3.2 Descriptive statistics and normality tests

Descriptive statistics and normality test results for core continuous variables are presented in Table 3. With the exception of resting heart rate in the badminton group, all other variables-including total high-altitude acclimatization score, total sleep score, blood oxygen saturation, vital capacity, and total physical activity-deviated significantly from normality (*p* < 0.001). Given the overall non-normal distribution of the dataset, results are presented as median (interquartile range) for non-normal variables.

### 3.3 Post-intervention group comparability analysis

Table 1 presents continuous variable comparisons; Table 4 further reports categorical variable comparisons (sex and ethnicity) using chi-square tests. Between-group comparisons at post-intervention assessment revealed highly significant differences in sex and ethnicity distribution between the basketball and badminton groups (*p* < 0.001). Among continuous variables, significant between-group differences were also observed for height, weight, resting heart rate, vital capacity, total PSQI score, and all PSQI subscale scores (*p* < 0.05). All the above variables were included as covariates in subsequent regression models to minimize the influence of initial between-group differences on intervention effect estimates.

**Table 4:** Between-group comparisons of categorical initial characteristics (*N* = 295).

Variable	Group	*n* and composition	χ^2^	*p*-value
Sex	Basketball	94 males (64.38%), 52 females (35.62%)	19.134	<0.001
	Badminton	58 males (38.93%), 91 females (61.07%)		
Ethnicity	Basketball	108 Tibetans (73.97%), 38 others (26.03%)	11.538	<0.001
	Badminton	82 Tibetans (55.03%), 67 others (44.97%)		

### 3.4 Correlation analysis

The results of correlation analysis for core variables are presented in Table 5. The total high-altitude acclimatization score was significantly positively correlated with total PSQI score (*r* = 0.213, *p* < 0.01) and resting heart rate (*r* = 0.184, *p* < 0.01), and significantly negatively correlated with total physical activity (*r* = −0.149, *p* < 0.05). Exercise type was significantly positively correlated with total PSQI score (*r* = 0.165, *p* < 0.01), and significantly negatively correlated with resting heart rate and vital capacity (*r* = −0.126, −0.165, *p* < 0.05). These correlations provided preliminary support for the hypothesized relationships, particularly the association between higher physical activity and better adaptation, and between poorer sleep quality and lower adaptation.

**Table 5:** Spearman rank correlation matrix of core variables.

Variable	1. Exerci-se type	2. Total high-altitude acclimatization score	3. Total PSQI sleep score	4. Total physical activity	5. Resting heart rate	6. Blood oxygen saturation	7. Vital capacity
1. Exercise type	1.000	0.093	0.165	−0.076	−0.126	0.076	−0.165
2. Total high-altitude adaptati-on score	-	1.000	0.213	−0.149	0.184	0.081	−0.026
3. Total PSQI sleep score	-	-	1.000	−0.041	0.083	0.018	−0.060
4. Total physical activity	-	-	-	1.000	0.028	−0.203	0.027
5. Resting heart rate	-	-	-	-	1.000	0.097	0.001
6. Blood oxygen saturation	-	-	-	-	-	1.000	0.051
7. Vital capacity	-	-	-	-	-	-	1.000

*p* < 0.05, *p* < 0.01 (two-tailed test); Exercise type coding: Basketball group = 1, Badminton group = 2.

### 3.5 Moderation effect of Total physical activity

First, the main effect of exercise type on high-altitude acclimatization was examined (Table 6). After adjusting for covariates, no significant main effect was observed (B = −0.6061, *p* = 0.5195). Next, the moderating effect of total physical activity was tested (Table 7). The interaction term between exercise type and total physical activity was significant (B = −2.9965, 95% CI [−5.1079, −0.8852], *p* = 0.0056), indicating a significant moderating effect. The overall model was significant (*R*^2^ = 0.173, *F* = 14.306, *p* < 0.001), explaining approximately 17.3% of the variance in acclimatization scores.

**Table 6:** Regression results for the main effect of exercise type on total high-altitude acclimatization score.

Variable	B	Robust SE	95% CI	*p*-value
Intercept	15.8066	16.0385	[−15.7614, 47.3747]	0.3252
Exercise type	−0.6061	0.9399	[−2.4561, 1.2439]	0.5195
Sex	2.1278	1.4912	[−0.8073, 5.0630]	0.1547
Ethnicity	6.3621	1.0544	[4.2866, 8.4375]	<0.001

Model fit: *R*^2^ = 0.155, *F* = 7.981, *p* < 0.001. The model controlled for the following covariates: sex, ethnicity, age, BMI, resting heart rate, height, weight, and vital capacity. Only results for key variables are presented in the table.

**Table 7:** Regression results for the moderating effect of total physical activity.

Variable	B	Robust SE	95% CI	*p*-value
Intercept	19.4670	9.3093	[1.1438, 37.7902]	0.0374
Exercise type	−0.4338	0.8987	[−2.2026, 1.3350]	0.6297
Total physical activity	4.9121	2.1323	[0.7151, 9.1090]	0.0220
Exercise type × Total physical activity	−2.9965	1.0727	[−5.1079, −0.8852]	0.0056

Model fit: *R*^2^ = 0.173, F = 14.306, *p* < 0.001. The model controlled for the following covariates: sex, ethnicity, age, BMI, resting heart rate, height, weight, and vital capacity. Only key variable results are presented in the table.

The Johnson–Neyman interval test revealed that the negative predictive effect of exercise type on high-altitude acclimatization became statistically significant (*p* ≤ 0.05) only when the standardized total physical activity score was ≥ 0.6151 (accounting for 13.90% of the sample). No significant between-group difference in high-altitude acclimatization score was observed at low or moderate activity levels.

Given the scoring rule of the high-altitude acclimatization questionnaire (higher scores indicate poorer adaptation), at low and moderate activity levels, no statistically significant difference in high-altitude acclimatization was observed between the badminton and basketball groups. At high activity levels, the badminton group showed significantly lower high-altitude acclimatization scores than the basketball group, indicating better adaptation.

**Figure 1:**
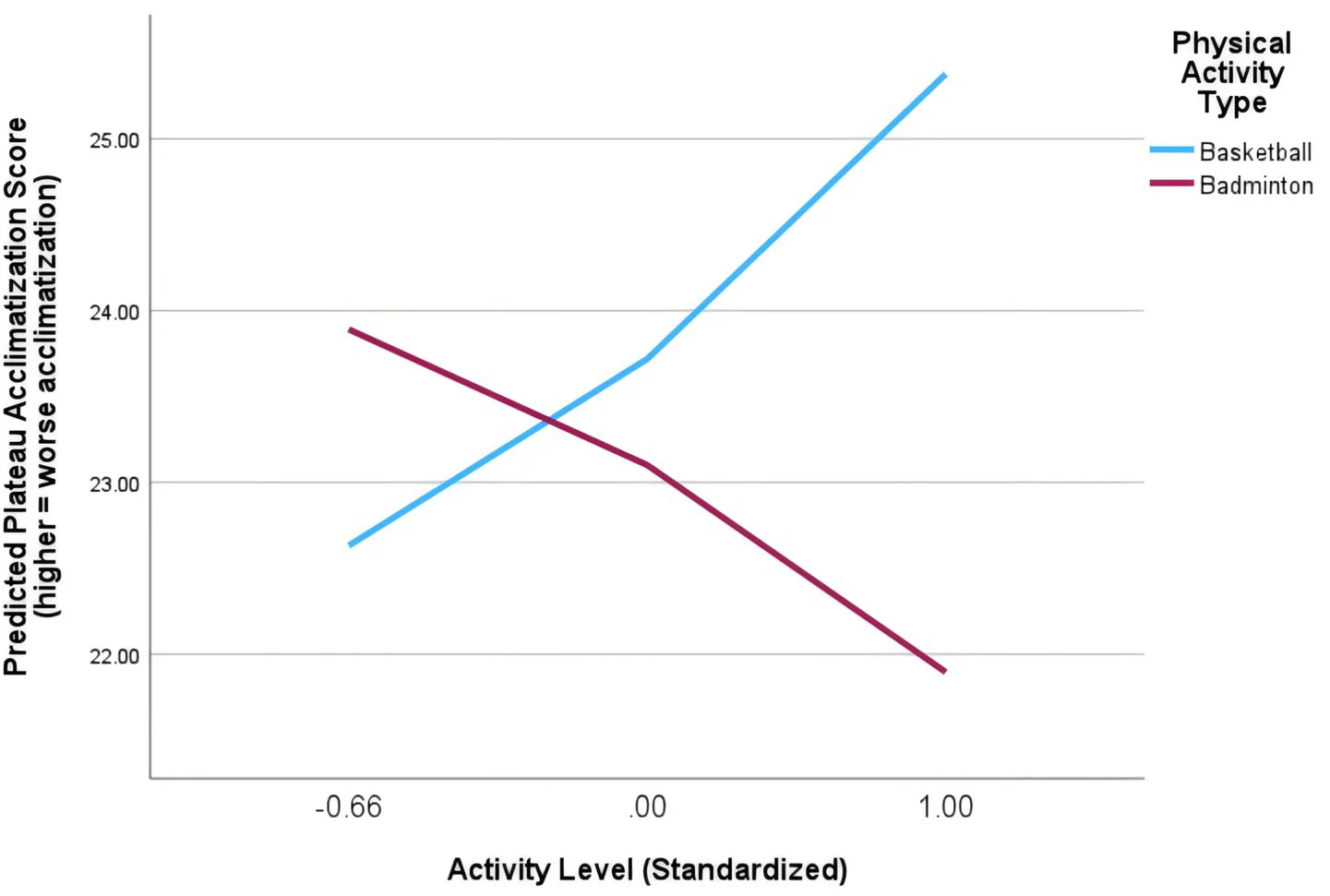
Moderating effect of total physical activity on the relationship between exercise type and high-altitude acclimatization (simple slope plot). Higher scores on the high-altitude acclimatization measure indicate poorer adaptation capacity. The figure shows the predictive effect of exercise type (basketball vs. badminton) on total high-altitude acclimatization score at different levels of total physical activity. Results indicate that as activity volume increases, the high-altitude acclimatization score of the basketball group gradually exceeds that of the badminton group, and the effect of exercise type becomes significant at high activity levels.

The simple slope plot is presented in Figure 1.

### 3.6 Mediation analysis of sleep quality

Given that the High-Altitude Adaptation Questionnaire contains sleep-related items (e.g., sleep quality at altitude), which may lead to content overlap with the mediating variable (PSQI total score), the following mediation analysis should be considered exploratory, and the results interpreted with caution. To examine the potential mediating role of sleep quality in the relationship between exercise type and high-altitude adaptation, the PROCESS macro (Model 4) was used with 5,000 bootstrap iterations. Results showed that the predictive effect of exercise type on the total PSQI score was not significant (B = 0.4255, *p* = 0.1595); therefore, the prerequisite for formal mediation testing was not met. The 95% bootstrap confidence interval for the indirect effect included zero ([−0.0877, 0.5635]), indicating that sleep quality did not show a significant mediating effect under the present intervention conditions. Furthermore, separate tests for each PSQI component (subjective sleep quality, sleep latency, sleep duration, habitual sleep efficiency, sleep disturbances, use of sleep medication, and daytime dysfunction) also revealed that the 95% bootstrap confidence intervals for indirect effects of all subscales included zero and were not statistically significant. These findings confirm that, in this sample, sleep quality did not play a significant mediating role in the relationship between exercise type and high-altitude adaptation (see Table 8).

**Table 8:** Results of the mediation effect test for total PSQI sleep score.

Path	Effect size (B)	Bootstrap SE	t	*p*-value	95% bootstrap CI
Total effect (Exercise type → High-altitude adaptation)	−0.4063	0.9174	−0.4428	0.6582	[−2.2119, 1.3994]
Direct effect (Exercise type → High-altitude adaptation)	−0.6145	0.9114	−0.6743	0.5007	[−2.4084, 1.1794]
Indirect effect (Exercise type → Total PSQI → High-altitude adaptation)	0.2083	0.1649	-	-	[−0.0877, 0.5635]

### 3.7 Supplementary analysis of sex confounding

To evaluate the potential influence of the significant sex imbalance in initial characteristics (see Table 1 for distribution) on the core conclusions, two supplementary analyses were conducted as pre-specified in Section 2.4. First, an exercise type × sex interaction test showed that the interaction term had no statistically significant predictive effect on total high-altitude acclimatization score (B = 0.5074, 95% CI [−3.0056, 4.0204], *p* = 0.7764), indicating no significant moderating effect of sex. Second, sex-stratified subgroup analysis revealed that in both the male (*n* = 152) and female (*n* = 143) subgroups, the pattern of the core moderating effect was consistent with that in the total sample, but neither reached statistical significance (*p* = 0.1198 and 0.0899, respectively). This pattern suggests that the core conclusions were not biased by sex distribution differences, and the non-significance in stratified analyses likely reflects reduced statistical power due to smaller subgroup sample sizes (see Tables 9, 10).

**Table 9:** Regression results for the exercise type × sex interaction effect test.

Variable	*B*	Robust SE	95% CI	*p*-value
Intercept	20.8900	11.2998	[−1.3506,43.1307]	0.0655
Exercise type	−1.1677	2.7675	[−6.6147,4.2793]	0.6734
Sex	1.2901	2.9295	[−4.4759,7.0561]	0.6600
Exercise type × Sex	0.5074	1.7848	[−3.0056,4.0204]	0.7764

Model fit: *R*^2^ = 0.145, F = 8.853, *p* < 0.001.

**Table 10:** Results of subgroup moderating effect tests stratified by sex.

Variable	Male subgroup (*n* = 152), *B* (SE)	*p*-value	Female subgroup (*n* = 143), B (SE)	*p*-value
Exercise type	−0.3872 (1.2104)	0.7496	−0.2984 (1.0247)	0.7713
Total physical activity	3.8762 (2.5641)	0.1327	3.2145 (2.0132)	0.1126
Exercise type × Total physical activity	−2.1147 (1.3521)	0.1198	−1.8762 (1.0983)	0.0899
Model fit	*R*^2^ = 0.168, *p* < 0.001	-	*R*^2^ = 0.159, *p* < 0.001	-

### 3.8 Robustness check

The robustness of the core findings was further verified by comparing between-group differences in physiological indicators before and after covariate adjustment. Without adjustment, the basketball group performed significantly better than the badminton group in resting heart rate and vital capacity (*p* < 0.05); however, after controlling for sex, ethnicity, age, BMI, height, and weight, these differences were no longer significant (*p* > 0.05). This indicates that the initial between-group differences in physiological indicators were mainly driven by initial confounding factors. After controlling for the 6 pre-specified demographic and anthropometric covariates (sex, ethnicity, age, BMI, height, and weight), between-group differences in resting heart rate and vital capacity were no longer statistically significant (*p* > 0.05).

## 4 Discussion

### 4.1 Core findings and consistency with research hypotheses

Based on a post-test only cluster-randomized controlled design, this study systematically tested three pre-specified hypotheses with clear and targeted results:

1. No significant difference was found between the two exercise types in their effects on sleep quality, and sleep quality did not mediate the relationship between exercise type and high-altitude acclimatization (Hypotheses 1 and 2 were not supported).
2. The differential effects of basketball and badminton on high-altitude acclimatization depended on the moderation of total physical activity (Hypothesis 3 was supported).
3. Initial sex confounding did not distort the core effects, and the results showed good robustness.

This conclusion moves beyond the traditional “single effect” framework of high-altitude exercise research, reveals the conditional mechanism of exercise affecting high-altitude adaptation, and provides empirical support for personalized intervention.

#### 4.1.1 Moderating effect of total physical activity: a “loading threshold” for high-altitude exercise interventions

This study confirmed the threshold moderating effect of total physical activity on the relationship between basketball/badminton intervention and high-altitude acclimatization among college students in the high-altitude region (interaction B = −2.9965, *p* = 0.0056), with the effect of exercise type reaching significance only when the standardized total physical activity was ≥ 0.6151 (accounting for 13.90% of the sample). This “load threshold effect” can be explained by the physiological limits of hypoxic adaptation and the distinct metabolic demands of the two sports. In the high-altitude environment, the body relies on improving cardiac output and oxygen utilization efficiency to enhance acclimatization. However, the distinct load characteristics of the two exercise modes suggest a critical difference in their impact on oxygen balance and physiological strain. Basketball sessions involved sustained high-intensity effort, which, given the reduced oxygen partial pressure at 3650 m, likely pushed the cardiovascular system toward its upper limit of aerobic capacity. This prolonged high demand may force the body to rely increasingly on anaerobic energy pathways, contributing to cumulative physiological strain. This excessive burden may counteract the potential benefits of exercise on acclimatization. Conversely, badminton’s intermittent pattern, characterized by alternating bursts of activity and brief recovery periods, inherently allowed for partial oxygen replenishment and metabolic waste clearance. Thus, badminton provided a potent aerobic stimulus without overwhelming the body’s compensatory mechanisms. While basketball’s sustained high-load characteristics may lead to excessive oxygen consumption and metabolic stress in a hypoxic environment, badminton’s intermittent skill-based load delivered effective aerobic stimulation while mitigating these risks. Critically, at low to moderate activity levels, the physiological stimulation of both sports remained within the body’s compensatory range, so the difference between the groups was not significant; at high activity levels, the cumulative strain induced by basketball exceeded the threshold of physiological compensation, whereas badminton’s intermittent nature allowed it to remain within the “therapeutic window,” thereby demonstrating superior acclimatization benefits, which is highly consistent with the findings of Altulea et al. (n.d.).

Importantly, these findings generalize across both indigenous Tibetan and migrant college students at high altitudes (~3,650 m). Given that the basketball group sustained heart rates of 130–150 bpm, a range approaching the estimated aerobic ceiling at 3650 m, the continuous metabolic demand likely challenged the participants’ cardiopulmonary reserves. Conversely, the intermittent nature of badminton, which fluctuated between 100–120 bpm during low-intensity phases and 140–160 bpm during intense rallies, inherently incorporated active recovery phases, which may have facilitated partial oxygen replenishment and mitigated excessive sympathetic activation typical of high-altitude exertion.

#### 4.1.2 Non-significant mediating effect of sleep: special features of the high-altitude environment and intervention dosage considerations

Unlike the “exercise → sleep → health” mediating pathway generally confirmed in normoxic low-altitude studies, previous studies have even found that nocturnal endurance exercise under hypoxic conditions impairs sleep quality to a greater extent. Sleep quality (total score and each component) did not play a mediating role in this study (Kobayashi et al., 2025). This may be because the predictive effect of exercise type on sleep quality was not significant (B = 0.4255, *p* = 0.1595) (Hoshikawa et al., 2013; Hrozanova et al., 2021). This difference may stem from the unique interference of the high-altitude hypoxic environment:

1. Low oxygen partial pressure may directly inhibit the sleep-regulating center in the ventrolateral preoptic area of the brainstem, reducing the proportion of slow-wave sleep and fragmenting sleep cycles; the intensity of this environmental interference may exceed the sleep-promoting effects of exercise.
2. The intervention dosage in this study (90 min per week) did not reach the “effective threshold” for improving sleep at high altitude. Normoxic low-altitude studies have shown that moderate-intensity exercise of ≥150 min per week can significantly improve sleep efficiency, whereas in a high-altitude environment, hypoxia-induced oxidative stress and autonomic nervous system dysregulation may require a higher exercise dosage and a longer intervention period.
3. The significant positive correlation between sleep quality and high-altitude acclimatization (r = 0.213, *p* < 0.01) indicates that sleep remains an important factor related to high-altitude acclimatization (Tan et al., 2024), however, it did not emerge as a mediating pathway through which exercise type affected high-altitude adaptation, suggesting the possible existence of an alternative pathway: “exercise → cardiopulmonary function → high-altitude adaptation”.

### 4.2 Integration and innovation relative to existing research

#### 4.2.1 Expanding the boundary conditions of exercise adaptation at high altitude

Previous high-altitude exercise research mostly focused on the main effect of a single exercise type, confirming that long-distance running, hiking, and other sports can promote adaptation by increasing hemoglobin concentration and improving respiratory muscle function (Hrozanova et al., 2021; Kobayashi et al., 2025), but did not pay attention to the differential effect of exercise type and moderating factors (Zhang et al., 2022; Zhang Y. et al., 2025).

#### 4.2.2 Revealing the unique nature of the exercise–sleep relationship at high altitude

In normoxic low-altitude studies, regular exercise prolongs deep sleep time by regulating autonomic nerve balance (reducing sympathetic nerve excitability and increasing vagus nerve tension), but this result was not replicated in this study. This discrepancy underscores the critical role of environmental context and population characteristics. Previous studies conducted at sea level involve populations without the confounding factor of chronic hypoxia, where exercise-induced autonomic regulation is the dominant pathway affecting sleep. In contrast, our participants were low-altitude migrant college students who had resided at high altitude for only approximately one year. Their bodies had not yet established stable hypoxic compensatory mechanisms (such as the enhanced ventilatory sensitivity seen in lifelong Tibetan residents). For this vulnerable population, the metabolic perturbation caused by exercise may act as an additional stressor on top of the existing hypoxic stress, potentially exacerbating autonomic nervous system dysregulation rather than promoting relaxation. This is consistent with the research findings of Liu et al. (2014) at an altitude of 3,700 m, which demonstrated that systolic blood pressure (SBP) and pulse pressure were higher following high-altitude exercise. The observed elevation in blood pressure in that study suggests a heightened cardiovascular response to hypoxic stress, which may contribute to difficulties in sleep onset and maintenance. Furthermore, compared to indigenous plateau residents who have developed stable physiological adaptations to chronic hypoxia, the migrant population in this study may not have fully established such compensatory mechanisms, rendering them more susceptible to the negative effects of hypoxia on sleep quality, a core reason for the non-significant sleep effects observed in our data.

### 4.3 Strengths and limitations

#### 4.3.1 Strengths

1. Rigorous cluster-randomized controlled design: this study adopted a rigorous cluster-randomized design with consistent instructors, intervention duration, and environmental conditions, effectively controlling non-treatment confounders.
2. Appropriate and robust statistical methods: the use of appropriate statistical methods-including HC3 robust standard errors for non-normal data, 5,000 bootstrap samples for confidence interval estimation, and Johnson–Neyman interval testing for moderation effects-enhances the reliability of findings.
3. Comprehensive confounding control strategy: comprehensive confounding control with eight covariates, supplemented by interaction and stratified analyses, reduces confounding bias.
4. Standardized and validated measurement tools: standardized measurement using validated scales (PSQI, IPAQ) and objective physiological indicators ensures data quality.
5. Rigorous sex confounding control and result robustness: regarding the sex imbalance observed at post-intervention assessment, we implemented a three-tier control strategy: (1) including sex as a covariate in all models, (2) testing the exercise type × sex interaction (*p* = 0.7764), and (3) conducting sex-stratified subgroup analyses. The results consistently indicated that sex did not confound the core findings. Furthermore, sensitivity analysis of physiological indicators showed that the original between-group differences in resting heart rate (*p* = 0.027) and vital capacity (*p* = 0.005) completely disappeared after controlling for covariates (*p* > 0.05), verifying the effectiveness of our confounding control strategy. All core results remained statistically consistent after adjustment, confirming that post-intervention group imbalances did not introduce meaningful bias into our findings. This ensures good internal validity of the conclusions.

#### 4.3.2 Limitations

1. Post-test only quasi-experimental design: this study employed a post-test only cluster-randomized quasi-experimental design. Although covariate adjustment and multiple sensitivity analyses were used to reduce potential confounding from between-group differences, residual confounding from unmeasured variables cannot be fully excluded. Therefore, the findings should be interpreted cautiously in terms of causal inference. Future studies should employ a pre-test-post-test design with comprehensive baseline assessments to further strengthen internal validity.
2. Limited generalizability of the single-center sample: the single-center sample included only college students from one university in Xizang, limiting generalizability to middle-aged, elderly, or long-term migrant plateau populations.
3. Insufficient intervention dosage for sleep improvement: the intervention dosage (90 min per week) may not have reached the effective threshold for sleep improvement at high altitude, and long-term effects beyond the 18-week intervention period remain unknown.
4. Self-reported sleep measurement bias: sleep assessment relied on self-reported PSQI without objective monitoring, which may introduce assessment bias. Additionally, sleep-related item overlap between the outcome scale and PSQI may cause measurement bias in the mediation analysis, which we addressed by treating all sleep-related mediation analyses as exploratory with no definitive causal inferences drawn.
5. Incomplete exploration of mediating mechanisms: potential mediators such as cardiopulmonary function, oxidative stress markers, and autonomic nervous system activity were not examined, leaving the full mechanism of exercise effects on high-altitude acclimatization incompletely revealed.
6. Limitations of heart rate-based load characterization: we acknowledge that using heart rate alone to characterize training intensity and load has inherent scientific limitations, as heart rate can be influenced by ambient temperature, emotional state, and individual autonomic nervous system reactivity independent of exercise load. Furthermore, continuous real-time heart rate monitoring was not conducted for all participants during the formal intervention due to equipment, funding, human resources, and operational constraints of large-scale public physical education teaching, preventing precise quantification of individual exercise load.
7. Additional exercise load and measurement biases: beyond the above limitations of heart rate monitoring, two additional sources of exercise load and measurement bias warrant discussion: (1) Despite strict adherence to the standardized syllabus, inherent load variability still existed between sessions and participants due to differences in skill proficiency, motivation, and voluntary effort during drills and match play. This intra-intervention load heterogeneity may introduce residual confounding in intervention effect estimates. (2) Habitual physical activity outside structured sessions was assessed via self-reported IPAQ-LF rather than objective accelerometry, which may carry recall bias and cannot accurately capture fine-grained differences in activity volume and intensity.

### 4.4 Future research directions

Several directions warrant further investigation. First, the threshold effect of total physical activity should be validated across diverse populations (e.g., middle-aged and older adults) and at different altitudes. Second, future studies should incorporate objective sleep measures (e.g., polysomnography) and examine cardiopulmonary function and oxidative stress markers as potential mediating pathways. Third, dose–response studies are needed to determine the optimal exercise dosage for concurrently improving acclimatization and sleep quality at high altitude.

## 5 Conclusion

This study provides the first head-to-head comparison of two popular team sports for high-altitude acclimatization and establishes total physical activity’s threshold effect as the core boundary condition for exercise intervention efficacy. No single exercise type is universally superior at 3650 m: badminton outperforms basketball only above a specific activity threshold, while both yield equivalent benefits for low-to-moderately active individuals. Furthermore, the well-established “exercise-sleep-health” pathway from sea level does not translate to high altitudes under a 90-min/week structured regimen, challenging direct extrapolation of low-altitude guidelines to plateau populations. All conclusions are supported by robust analyses controlling for demographic and physiological confounders.

These findings have three key practical implications: First, highly active students should prioritize intermittent sports like badminton to avoid cumulative hypoxic strain from sustained high-intensity exercise such as basketball. Second, low-to-moderately active students may choose either sport based on preference, as adherence is the primary determinant of acclimatization benefits. Third, standalone weekly physical education classes are insufficient to address high-altitude sleep disturbances, necessitating complementary strategies such as sleep hygiene education or relaxation training.

Scientifically, this study breaks the traditional “one-size-fits-all” paradigm in high-altitude exercise research by demonstrating context-dependent intervention efficacy. Personalized exercise programs for plateau populations must account for individual habitual activity levels. Future research should validate these findings across diverse populations and explore optimal exercise dosages for improving both acclimatization and sleep at high altitude.

## Data Availability

The datasets presented in this article are not readily available because data sharing is not applicable due to regional research regulations and ethical restrictions in Xizang. The dataset involves sensitive geographical and population information, and public release or external distribution is prohibited by local regulatory requirements. Requests to access the datasets should be directed to Mingli Xi, 2495139411@qq.com.
