# Correction: Effects of exercise type and total physical activity on high-altitude acclimatization among college students

**DOI:** 10.3389/fpsyg.2026.1958264

**Published:** 2026-09-01

**Authors:** Mingli Xi, Changhao Tian, Yonghao Ou, Runqing Lu, Chen Wang, Hao Li

**Affiliations:** College of Education, Xizang University, Xizang Autonomous Region, Lhasa, China

**Keywords:** exercise therapy, high altitude acclimatization, high altitude adaptation, moderating effect, physical activity, sleep quality

Author Mingli Xi was erroneously listed as co-corresponding author. The sole corresponding author should be Hao Li. All author orders remain unchanged. The original version of this article has been updated.

